# Validation of HR-pQCT against micro-CT for morphometric and biomechanical analyses: A review

**DOI:** 10.1016/j.bonr.2020.100711

**Published:** 2020-08-24

**Authors:** Nicholas Ohs, Caitlyn J. Collins, Penny R. Atkins

**Affiliations:** a Institute for Biomechanics, ETH Zurich, Zurich, Switzerland; b Department of Osteoporosis, Inselspital, Bern, Switzerland

**Keywords:** HR-pQCT, Micro-CT, Patient evaluation, Bone morphometrics, Finite element analysis, Segmentation

## Abstract

High-resolution peripheral quantitative computed-tomography (HR-pQCT) has the potential to become a powerful clinical assessment and diagnostic tool. Given the recent improvements in image resolution, from 82 to 61 μm, this technology may be used to accurately quantify in vivo bone microarchitecture, a key biomarker of degenerative bone diseases. However, computational methods to assess bone microarchitecture were developed for micro computed tomography (micro-CT), a higher-resolution technology only available for ex vivo studies, and validation of these computational analysis techniques against the gold-standard micro-CT has been inconsistent and incomplete. Herein, we review methods for segmentation of bone compartments and microstructure, quantification of bone morphology, and estimation of mechanical strength using finite-element analysis, highlighting the need throughout for improved standardization across the field.

Studies have relied on homogenous datasets for validation, which does not allow for robust comparisons between methods. Consequently, the adaptation and validation of novel segmentation approaches has been slow to non-existent, with most studies still using the manufacturer's segmentation for morphometric analysis despite the existence of better performing alternative approaches. The promising accuracy of HR-pQCT for capturing morphometric indices is overshadowed by considerable variability in outcomes between studies. For finite element analysis (FEA) methods, the use of disparate material models and FEA tools has led to a fragmented ability to assess mechanical bone strength with HR-pQCT. Further, the scarcity of studies comparing 62 μm HR-pQCT to the gold standard micro-CT leaves the validation of this imaging modality incomplete.

This review revealed that without standardization, the capabilities of HR-pQCT cannot be adequately assessed. The need for a public, extendable, heterogeneous dataset of HR-pQCT and corresponding gold-standard micro-CT images, which would allow HR-pQCT users to benchmark existing and novel methods and select optimal methods depending on the scientific question and data at hand, is now evident. With more recent advancements in HR-pQCT, the community must learn from its past and provide properly validated technologies to ensure that HR-pQCT can truly provide value in patient diagnosis and care.

## 1 Introduction

Micro computed tomography (micro-CT) was first used to analyse the trabecular structure of bone in three dimensions in 1989 (Feldkamp et al., 1989). Shortly thereafter, the first commercial micro-CT device was made available (Rüegsegger et al., 1996); this quickly led to widespread adoption of micro-CT as a standard research tool for bone tissue analysis at the micrometre (μm) scale. In contrast to two-dimensional (2D) methods, the three-dimensional (3D) acquisition capabilities and high resolution (up to 1 μm) allowed for direct assessment of 3D structures. By 2008, the plethora of commonly used micro-CT technologies and bone morphology analysis techniques with translational applications warranted a comprehensive book chapter (Stauber and Müller, 2008). More recently, micro-CT-based finite element analysis (FEA) has provided an experimentally validated method to assess bone mechanical strength and failure non-invasively (Chen et al., 2017; Hambli, 2013).

**Fig. 1:**
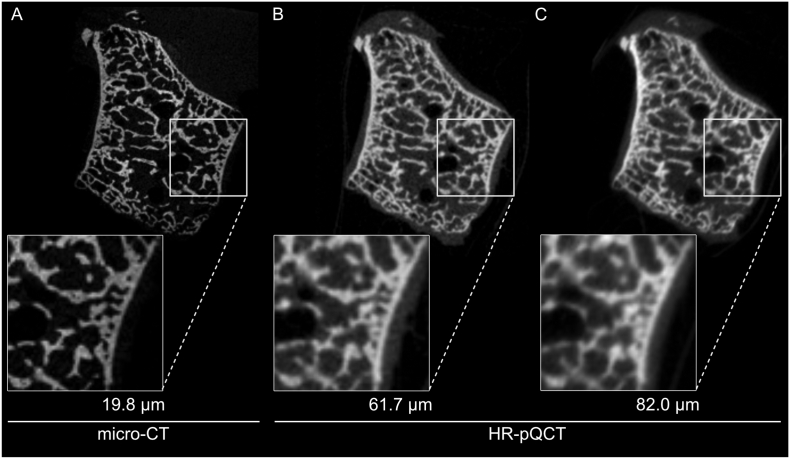
With increasing voxel size, pores and edges of smaller features become difficult to capture using computed tomography techniques. In the reconstructed (A) micro-CT, (B) 61 μm HR-pQCT, and (C) 82 μm HR-pQCT images of an ex vivo trapezium, extracted from the wrist of a patient with severe arthritis, pores near the cortex become hard to distinguish with decreasing image resolution. Adapted from (Mys et al., 2019).

In parallel with the laboratory development of micro-CT, peripheral quantitative computed tomography (pQCT) emerged as a potential method for identifying risk factors of disease, such as osteoporosis (Müller et al., 1989). Given that existing clinical tools, such as dual energy x-ray absorptiometry (DXA), were unable to adequately identify patient at risk of fracture, pQCT provided an enhanced method to evaluate bone clinically (Bolotin and Sievänen, 2001; Järvinen et al., 2008; Nelson et al., 2002). While the resolution of pQCT is lower than micro-CT (170 μm voxels), it enabled patient imaging and non-invasive, time-lapse patient studies. Comparisons with micro-CT measurements were, however, limited due to the order of magnitude difference in image resolution. Moreover, the resolution of pQCT prohibited accurate assessment of bone microarchitecture for patients with degenerative bone diseases, such as osteoporosis, where the average thickness of individual trabeculae is 200 μm (Borah et al., 2004, Borah et al., 2006). To combat this, high-resolution pQCT (HR-pQCT) devices were introduced as improved successors to pQCT devices. These devices (XtremeCT I and II, Scanco Medical) have isotropic voxel sizes of 82 and 61 μm, respectively, which has allowed for direct comparison to micro-CT (Fig. 1). Unfortunately, the conclusions of these studies have been inconsistent, potentially due to the varied methods of calculation for the parameters used for comparison of HR-pQCT images to micro-CT images.

Since HR-pQCT devices are now available and in use in many clinical centres, the establishment of image processing and analysis standards are crucial for comparisons between studies and research groups. Therefore, we aim to provide a comprehensive review of existing computational processing and analysis tools which have been validated against the micro-CT gold standard for HR-pQCT and highlight the need for improved standardization across the field.

## 2 Data

For this purpose, the keywords HR-pQCT and micro-CT were searched on PubMed. Due to automatic term mapping, this included an extensive list of closely related terms. Of the 68 papers identified, 31 provided data comparing HR-pQCT and micro-CT (Table 1). The most common exclusion criterion was the lack of micro-CT data, despite the use of the keyword in the paper. Of the selected studies, 25 evaluated only 82 μm (first generation) HR-pQCT, and one study evaluated both 82 μm and 61 μm HR-pQCT (Fig. 1). The remaining five studies looked at both HR-pQCT device resolutions, but emulated one or both resolutions by acquiring lower-resolution images on a higher-resolution device or by scaling down higher-resolution images in post-processing (Table 1). Studies that utilized 82 μm images, either emulated or from XtremeCT I, are referred to as 82 μm HR-pQCT studies, while studies that utilized 61 μm images, either emulated or from XtremeCT II, are referred to as 61 μm HR-pQCT studies.

**Table 1:** HR-pQCT image modalities, anatomical sites, and reported disease status or other classification of samples used in validation studies.

Study	82 μm	61 μm	Anatomical site(s)	Sample classification (as applicable)
(Buie et al., 2007)	Yes	No	Radius/tibia	–
(Burghardt et al., 2007)	Yes	No	Femoral Head	Degenerative joint disease
(MacNeil and Boyd, 2007)	Yes	No	Radius	–
(Varga and Zysset, 2009)	Yes	No	Radius	–
(Cohen et al., 2010)	Yes	No	Radius/tibia/iliac crest^a^	Idiopathic osteoporosis/hypoparathyroidism
(Liu et al., 2010)	Yes	No	Tibia	–
(Nishiyama et al., 2010)	Yes	No	Radius	Osteopenia
(Liu et al., 2011)	Yes	Emulated^b^	Tibia	–
(Pahr et al., 2012)	Yes	No	Vertebrae	Fracture
(Tjong et al., 2012)	Yes	Emulated^c^	Radius	–
(Liu et al., 2013)	Yes	No	Tibia	Fracture
(Zebaze et al., 2013)	Yes	No	Radius/tibia	–
(Krause et al., 2014)	Yes	No	Radius/tibia	Osteoporosis
(Ostertag et al., 2014)	Yes	No	Tibia	–
(Jorgenson et al., 2015)	Yes	No	Tibia	–
(Manske et al., 2015)	Yes/emulated^d^	Yes	Radius	–
(Christen et al., 2016)	Emulated^b^	Emulated^b^	Radius	–
(Klintström et al., 2016)	Yes	No	Radius	–
(Ostertag et al., 2016)	Yes	No	Tibia/femur	–
(Scharmga et al., 2016)	Yes	No	Finger	–
(Zhou et al., 2016)	Yes	No	Radius/tibia	–
(Hosseini et al., 2017)	Emulated^e^	Yes	Radius	–
(Peters et al., 2017)	Yes	No	Joints of index finger	–
(Werner et al., 2017)	Yes	No	Finger	Rheumatoid arthritis
(Alsayednoor et al., 2018)	Yes	No	Calcaneus	–
(Chiang et al., 2018)	Yes	No	Radius	Menopause
(Metcalf et al., 2018)	Yes	No	Calcaneus	–
(Ang et al., 2020)	Yes/no	No/yes	Radius/tibia	–
(Mys et al., 2019)	Yes	Yes	Trapezium	Severe arthritis
(Soltan et al., 2019)	Yes	No	Radius	–
(Wang et al., 2019)	Yes	No	Radius/tibia	–

a Scanned with micro-CT only.

b Downscaled micro-CT images.

c Scanned at 41 μm using a 82 μm HR-pQCT device in non-patient mode.

d Comparison to micro-CT only with emulated data.

e Scanned using a 61 μm HR-pQCT device.

All selected studies utilized human bone samples. While many studies evaluated a single anatomical site, eight studies analysed more than one anatomical site. In total, the radius was evaluated in 17 studies, the tibia in 11, the femur in two, various locations in the hand (trapezium or finger joints) in four, the calcaneus in two, the vertebrae in one, and the iliac crest in one (Table 1). Studies included data from healthy patients, as well as patients with idiopathic osteoporosis, idiopathic hypoparathyroidism, osteopenia, osteoporosis, previous fracture, severe arthritis, and degenerative joint diseases.

## 3 Segmentation

**Fig. 2:**
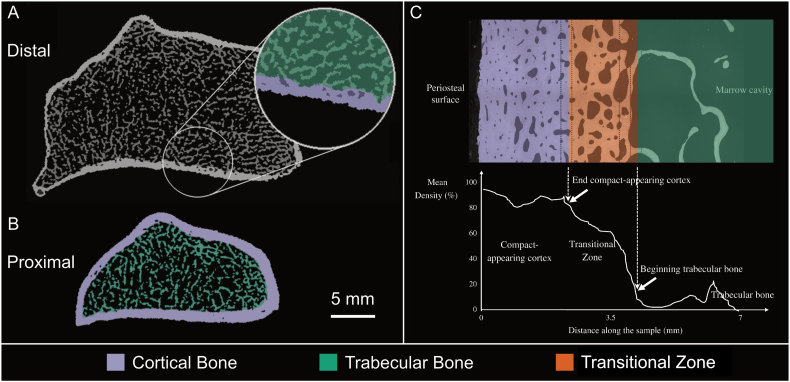
Micro-CT and HR-pQCT image segmentation algorithms predominantly target separation of the cortex and trabecular compartment. (A) The standard manufacturer approach for segmentation of HR-pQCT data was developed to identify the periosteal and endosteal margins of the distal radius (pictured) or tibia and separate the bone into two compartments: cortical (purple) and trabecular (green); (B) Quantification of bone microstructure in these compartments, for example the morphological assessment of individual trabeculae (highlighted in green) or cortical pores (black spots within the purple, cortical segmentation), can then be performed. Adapted from (Burghardt et al., 2010). (C) One alternative segmentation approach identifies an additional compartment, the transitional zone (orange), which is representative of the junction between cortical and trabecular compartments; here, intracortical remodelling can blur the line between cortical and trabecular compartments. Images in A and B were captured with 82 μm HR-pQCT, while the image in C was captured with 2.5 μm scanning electron microscopy. Adapted from (Zebaze et al., 2013).

Most micro-CT image analysis requires segmentation, which is the identification and partitioning of objects and boundaries of interest, as a pre-processing step. Only seven studies used a common segmentation procedure, while the remaining studies used different procedures for each imaging modality. Seventeen studies used segmentation to separate the cortical shell and the trabecular compartment (Fig. 2A) and 20 studies separated the individual trabeculae from the background (Fig. 2B). Despite the lack of an industry standard for micro-CT segmentation and the increased noise and blurring of lower resolution images (Fig. 1C), micro-CT segmentation methods were commonly used to develop and validate approaches for HR-pQCT.

### 3.1 Segmenting bone compartments

Trabecular and cortical compartments were segmented using the manufacturer's approach for HR-pQCT, the gold-standard approach micro-CT, and alternative approaches to segment both types of images.

#### 3.1.1 HR-pQCT – manufacturer's approach

The manufacturer segmentation procedures for HR-pQCT are widely used in HR-pQCT studies (Whittier et al., 2020). The approach for 82 μm HR-pQCT begins with a semi-automated hand-drawn contour of the periosteal surface, generated using the scanner manufacturer's software. From here, the trabecular bone compartment is separated from the cortical bone compartment. Herein, the voxel-based intensity data is converted to a physical density of hydroxyapatite (HA) (mg HA/cm^3^) and blurred into compartments of low- and high-density, generating a Gaussian-weighted mean image. A threshold of one-third of the apparent cortical bone density is then used to isolate the cortical and trabecular compartments. This segmentation procedure was implemented in a majority of the studies without modification.

The 61 μm HR-pQCT manufacturer segmentation procedure is based on a dual threshold technique and a cortical pore analysis to separate the cortex from the trabecular compartment (Fig. 2A) and was developed to avoid the introduction of operator error associated with hand-drawn contours. The dual threshold technique reduces the inclusion of noise by applying a higher value threshold to segment the higher-density cortex. Closing and connectivity operations are applied to generate the bone outer segmentation. This method works well provided there are no large gaps in the cortex, e.g. large Volkmann's canals, which disturb the connectivity operations. Large-radius blurring provides a smooth transition from the cortex into the trabecular compartment and a lower value threshold isolates the trabecular compartment. Before implementation into the manufacturer's software, the algorithm was applied to 82 μm HR-pQCT and micro-CT images and validated against the respective hand-drawn contours (Buie et al., 2007). The cortical pore analysis adds voids that are not connected to the background to the cortical compartment, as pores. The approach was validated against hand-drawn contours (Burghardt et al., 2010).

#### 3.1.2 Gold-standard micro-CT segmentation method

For micro-CT data, the cortical and trabecular compartments are separated using hand-drawn contours. However, semi-automatic procedures, including interpolating between two hand-drawn contours or snapping periosteal hand-drawn contours to nearby edges in an image, have been included in segmentation software packages to speed up the process of segmentation.

#### 3.1.3 Common segmentation using the software Fiji

Soltan and colleagues used Fiji (Schindelin et al., 2012) to generate a periosteal surface segmentation for cadaveric radii scanned with both 82 μm HR-pQCT and synchrotron radiation micro-CT (17.7 μm voxels) (Soltan et al., 2019). For both image modalities, the segmentation was generated using a threshold of 400 mg HA/cm^3^ and an optimized number of erosion iterations, such that the automatically generated HR-pQCT cortical masks most closely resembled the hand-drawn micro-CT contours.

#### 3.1.4 Common segmentation of the transitional zone

Zebaze and colleagues proposed a three-compartment segmentation method which produced cortical and trabecular compartments, as well as a transitional zone (Fig. 2C) (Zebaze et al., 2013). Through comparison to expert-generated hand-drawn contours of scanning electron microscopy images, their implementation produced accurate and reproducible cross-sectional areas of the three compartments for 82 μm HR-pQCT and micro-CT images. However, cortical interruptions greater than a few voxels, as would be expected for porous bone samples, resulted in method failure. For this reason, their analysis was limited to only 40 diaphyseal image slices, due to the reduced frequency of cortical interruptions in this region. Further, this segmentation method was implemented on 2D images, not on a 3D image volume, thus the application to consecutive slices would likely not result in a consistent and smooth surface, longitudinally. Importantly, Zebaze and colleagues both performed the validation study and co-wrote the patent behind the commercial software of this proposed method, StrAx1.0.

#### 3.1.5 Common segmentation with thickness-based separation

Due to the lack of clarity in the transition between the cortical and trabecular compartments in the epiphysis, Ang and colleagues modified the dual-threshold segmentation algorithm (Buie et al., 2007) to consistently segment images of both the diaphysis and the epiphyses (Ang et al., 2020). Here, a blurred distance measure of all slice-based pixels touching the outer surface segmentation was cropped at the outer surface to generate the cortical compartment segmentation. This algorithm was applied to relatively low-resolution micro-CT (50 μm) and HR-pQCT (both 61 and 82 μm) images and compared against the respective hand-drawn contours generated for each image.

### 3.2 Segmenting bone microstructure

For assessment of bone microstructure, the trabecular compartment is further segmented into bone and background. Since individual trabeculae can be only a few voxels thick for HR-pQCT images (Fig. 1), obtaining an accurate representation of the scanned bone structure can be challenging.

#### 3.2.1 HR-pQCT – manufacturer's approach

Laib and colleagues introduced a two-step procedure on pQCT image (165 μm), which has since been implemented in the manufacturer's software (Laib et al., 1998; Laib and Rüegsegger, 1999a). The image is first filtered and then a threshold is applied to isolate the trabecular structure. For 82 μm HR-pQCT image, an edge-enhancing Laplace-Hamming filter is used in combination with a threshold of 400 after normalization of the image to 1000 Hounsfield units. For 61 μm HR-pQCT image, a noise-reducing and structure enhancing Gaussian filter is used (sigma 0.8, filter 3x3x3) in combination with a 320 mg HA/cm^3^ threshold (Hosseini et al., 2017; Manske et al., 2015, Manske et al., 2017; Mys et al., 2019).

#### 3.2.2 Micro-CT segmentation methods

Micro-CT data is processed similarly to the 61 μm HR-pQCT images, however Gaussian settings and threshold values are varied. Gaussian sigma values ranged from 0.5 to 1.2 and filter sizes ranged from 3x3x3 to 5x5x5 (Table 2). With regards to threshold, some studies used specimen-specific thresholds given in percent of the maximum intensity value, while other studies used fixed thresholds given in mg HA/cm^3^ for all specimens. In more recent studies, starting in 2015, a fixed threshold in terms of mg HA/cm^3^ was more commonly used (Christen et al., 2016; Hosseini et al., 2017; Jorgenson et al., 2015; Manske et al., 2015; Soltan et al., 2019). Interestingly, none of the studies selected for this review used the same threshold, even when identical micro-CT devices were used (Table 2). When provided, the reason for choosing a particular threshold was either visual assessment (Burghardt et al., 2007; Manske et al., 2015; Metcalf et al., 2018), matching of morphometric parameters to the HR-pQCT scans (Peters et al., 2017), or being consistent with previous studies conducted using that dataset (Christen et al., 2016).

**Table 2:** Gaussian filter and threshold values used on gold standard micro-CT images.

Study	Morphometric analysis	CT device	Gaussian settings		Voxel size [μm]	Threshold
			Sigma	Support		
(Cohen et al., 2010)	Trabecular, Cortical	μCT 40	1.2	3x3x3	18	34% max
(MacNeil and Boyd, 2007)	Trabecular, Cortical	vivaCT 40	1.2	5x5x5	38	11.2% max
(Metcalf et al., 2018)	Trabecular	SkyScan 1172	1.2	5x5x5	17	33% max^a^
(Hosseini et al., 2017)	Trabecular	μCT 100	0.8	3x3x3	16	320 mg HA/cm^3^
(Burghardt et al., 2007)	Trabecular	μCT 40	–	–	16	25% max
(Manske et al., 2015)	Trabecular	XtremeCT II	0.8	3x3x3	30.3	390 mg HA/cm^3^
(Ostertag et al., 2014)	Cortical	SR Grenoble	–	–	7.5	53.9% max
(Ostertag et al., 2016)	Cortical	Skyscan 1172	–	–	7.5	15.6% max
(Chiang et al., 2018)	Cortical	vivaCT 40	–	–	19	960 mg HA/cm^3^
(Soltan et al., 2019)	Cortical	SR Canadian Light Source	–	–	17.7	400 mg HA/cm^3^
(Nishiyama et al., 2010)	Cortical	vivaCT 40	1.2	5x5x5	19	18.4% max
(Peters et al., 2017)	Cortical	μCT 80	0.8	3x3x3	18	24.7% max
(Alsayednoor et al., 2018)	Trabecular	SkyScan 1172	0.5	5x5x5	17.4	90^b^
(Christen et al., 2016)	Trabecular	vivaCT 80	1.2	3x3x3	25	450 mg HA/cm^3^
(Jorgenson et al., 2015)	Cortical	μCT 100	–	–	9	750 mg HA/cm^3^

Other terms: HA, hydroxyapatite; SR, synchrotron radiation.

a For select cases, the threshold was visually determined to be 35% of the maximum.

b Unknown units.

#### 3.2.3 Common segmentation methods for HR-pQCT and micro-CT

A histogram approach (Ridler and Calvard, 1978) was used on both micro-CT and 82 μm HR-pQCT images to separate bone from background using an image-specific, histogram-based threshold (Varga and Zysset, 2009). Alternatively, Hosseini and colleagues used the manufacturer based filtering for each image modality followed by a fixed global threshold of 320 mg HA/cm^3^ for all images (Hosseini et al., 2017).

#### 3.2.4 Alternative segmentation methods for HR-pQCT

Several additional segmentation methods have been developed specifically for either HR-pQCT or micro-CT images. One such strategy is that of local adaptive thresholds, which uses gradient edge detection to separate trabeculae from background in 82 μm HR-pQCT images (Burghardt et al., 2007). The algorithm analyses voxels at the edges of a scanned bone structure to preserve all trabeculae without filling small pores.

Another approach is to use an automated 3D region growing algorithm (ARG) (Revol-Muller et al., 2002) which has been compared to the manufacturer's default segmentation for 82 μm HR-pQCT images (Klintström et al., 2016). The region of interest is isolated using a very high threshold and then grown over multiple iterations using decreasing thresholds. The iteration that performed best with regards to an unspecified assessment function is chosen as the final segmentation. Importantly, this study used an alternative, Otsu histogram-based segmentation approach for their gold standard micro-CT images (Otsu, 1979), thus it is difficult to conclude whether the approach is accurate. Alsayednoor and colleagues (Alsayednoor et al., 2018) developed a method to segment 82 μm HR-pQCT images that preserved the underlying geometry of the bone structure according to fractal theory (Alberich-Bayarri et al., 2010). Therein, a geometry-preserving threshold that best matched the fractal dimension curves generated for each HR-pQCT and corresponding micro-CT image was chosen for each specimen, which is important for mechanical analysis (Alsayednoor et al., 2018). Notably, for morphometric analysis a threshold preserving the ratio of bone volume to compartment volume can be used instead.

### 3.3 Discussion

A variety of approaches have been shown to successfully segment aspects of the trabecular structure, the cortex, and the transitional zone. However, only one study compared the results of their proposed segmentation method on 61 μm HR-pQCT images directly to those of micro-CT (Ang et al., 2020). Importantly, the quality of segmentation was nearly always validated relative to morphometric measurements or finite element analysis results, instead of directly, which may not provide adequate validation. Furthermore, none of the studies included in this analysis attempted to validate their segmentation techniques on fractured bone or degenerative disease cases, such as osteoporosis or osteoarthritis.

Since none of the reviewed segmentation approaches were capable of segmenting cortices with larger gaps, an alternative approach, such as 3D active contours which can be tuned parametrically (Caselles et al., 1997; Hafri et al., 2016a, Hafri et al., 2016b; Kass et al., 1988; Marquez-Neila et al., 2014), might be necessary to provide accurate automatic segmentation for bones with reduced cortical connectivity. Due to the scope of this review, methods not yet validated against gold standard micro-CT, e.g. (Treece et al., 2010, Treece et al., 2012), have not been mentioned but might prove useful for the analysis of HR-pQCT images after validation.

Due to the limited validation of most approaches, a single method cannot be recommended without reservation. One of the greatest problems in identifying an optimal segmentation approach is the lack of available datasets for comparison. Currently, each study relies on a single, often homogenous, dataset for evaluation, while the availability of an extendable, publicly available, and diverse dataset of micro-CT and corresponding HR-pQCT images of various anatomical sites and disease states would allow for the direct comparison of the performance of different approaches. This would, in turn, allow for a clear recommendation as to which segmentation approach should be used for each type of data.

## 4 Morphometrics

Degenerative bone diseases not only alter the overall bone mass, but also the underlying microstructure of the bone (Brandi, 2009; Zhang et al., 2010). Hence, methods to quantitatively characterize bone microstructure (static parameters) and microstructural change over time (dynamic parameters) were developed for micro-CT (Goulet et al., 1994; Hildebrand et al., 1999; Hildebrand and Rüegsegger, 1997; Odgaard and Gundersen, 1993; Rüegsegger et al., 1996; Schulte et al., 2011; Wachter et al., 2001; Whitehouse, 1974). With the advent of HR-pQCT, these existing methods have been applied to HR-pQCT images and working alternatives have been investigated. In total, 25 of the selected studies evaluated the accuracy of morphometric parameters derived from HR-pQCT images relative to those derived from micro-CT images. Several of these parameters have been implemented in the manufacturer's device software, increasing their frequency within the literature.

Anatomical sites used for these morphometric comparisons included the radius (15 studies), tibia (10 studies), femoral head (one study), calcaneus (one study), and trapezium (one study). Generally, the median prediction accuracy was in the R^2^ range of 0.50 to 0.91 across all morphometric parameters. However, one study comparing 82 μm HR-pQCT images of the radius and tibia to micro-CT images of iliac crest biopsies reported lower morphometric prediction accuracy than all other evaluated studies (R^2^ of 0.02 to 0.27) (Cohen et al., 2010). The noticeable drop in prediction accuracy demonstrates the importance of anatomical site matching when performing such comparisons. Due to the difficulty in interpreting these results, the study by Cohen and colleagues has not been included in this Section. For studies including several anatomical sites, R^2^ values have been reported separately for each anatomical site.

### 4.1 Analysis methods

The compartmental segmentation gave rise to two separate classes of morphometric parameters: trabecular and cortical parameters. Parameters have been abbreviated based on standardized nomenclature (Parfitt et al., 2009).

#### 4.1.1 Regression analysis

Data are presented in the format of (median R^2^ value for 82 μm results, median R^2^ value for 61 μm results), unless otherwise noted. R^2^ values are described as good (R^2^ ≥ 0.80), moderate (0.60 ≤ R^2^ < 0.80), slight (0.40 ≤ R^2^ < 0.60), or poor (R^2^ < 0.4). When available, regression analysis values (slope and intercept) were extracted and converted to the form *slope* * value_HR-pQCT_ + *intercept* = value_micro-CT_, as necessary. Linear regressions were only reported when data from at least five studies was provided. The ideal regression parameters (slope = 1, intercept = 0) indicate perfect agreement between HR-pQCT and micro-CT results. Regression parameters deviating from this optimum indicate that the HR-pQCT results have to be calibrated to match those of micro-CT. Greater regression parameters (slope > 1, intercept > 0) indicate an underestimation of morphometric values by HR-pQCT, whereas lesser parameters (slope < 1, intercept < 0) indicate overestimation. For mixed parameters, over- and underestimation depend on the morphometric value.

#### 4.1.2 Indirect and direct methods

The analysis of morphometrics has been split into direct and indirect comparisons. Direct refers to the direct application of micro-CT algorithms, while indirect refers to morphometrics calculated using the recommended manufacturer's approach. While both methods were used for 82 μm HR-pQCT, only direct methods were used for 61 μm HR-pQCT. Similar to segmentation, the manufacturer's approach for morphometric parameter calculation is based on studies performed using pQCT devices. Since any modifications to these original methods by the manufacturer have not been published and can be made at any time without notice, a comprehensive overview of these methods can only be provided by the manufacturer.

### 4.2 Trabecular parameters

Since HR-pQCT images have increased noise and only resolve single trabeculae with a few voxels (Fig. 1B, C), a thorough validation of analysis methods against a high quality gold standard such as micro-CT is necessary.

#### 4.2.1 Bone volume fraction

**Fig. 3:**
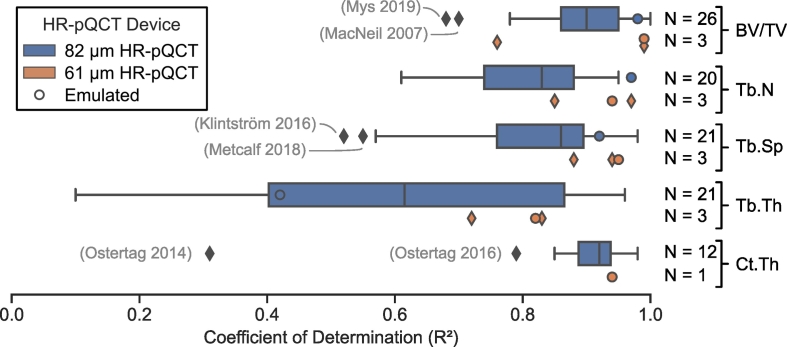
Coefficient of determination for the most commonly reported morphometric parameters, which were reported in more than ten studies: bone volume fraction (BV/TV), trabecular number (Tb.N), trabecular separation (Tb.Sp), trabecular thickness (Tb.Th), and cortical thickness (Ct.Th). Emulated image data, such as 82 μm HR-pQCT data obtained from downsampled micro-CT or 61 μm HR-pQCT devices, is included both in the box plot and additionally highlighted by circles to identify any differences to parameters derived from 61 μm or 82 μm HR-pQCT images using the default manufacturer settings. In general, we observe that BV/TV can be captured well with both HR-pQCT resolutions while results for other parameters, such as Tb.Th, vary drastically across studies for 82 μm images and should be interpreted with caution.

Bone volume fraction (BV/TV), the most commonly reported trabecular parameter, was captured well by both 82 μm and 61 μm HR-pQCT devices when compared to micro-CT (R^2^ = 0.91, 0.99) (Fig. 3). The lowest agreement with micro-CT was found for 82 μm HR-pQCT scans of the trapezium (Mys et al., 2019) (R^2^ = 0.68) (Fig. 3). Two approaches have been used to compute BV/TV for 82 μm HR-pQCT images; to date, the indirect density approach (Laib et al., 1998) has performed slightly better (R^2^ = 0.92, *N* = 12) than the direct segmentation-based approach (R^2^ = 0.88, *N* = 14) when comparing to BV/TV measured from the gold standard micro-CT. While both approaches agree well, neither is unassailable. The density-based approach suffers from inaccurate grey-values due to beam hardening effects, which disproportionately affect voxel intensities near the centre of the image. The segmentation-based approach suffers from partial volume effects, thus it is very sensitive to the chosen threshold (Varga and Zysset, 2009). The use of different threshold approaches on BV/TV yielded a range of R^2^ values from 0.88 to 0.95 when images of the same samples were compared with micro-CT (Varga and Zysset, 2009). The highest agreement was found for microstructural segmentation using a Laplace-Hamming filter followed by a fixed threshold of 40% of the maximum grey value. These settings resulted in the most visually similar bone architecture and connectivity relative to gold standard micro-CT. The local adaptive and the fixed threshold segmentation approaches showed equal agreement when comparing 82 μm HR-pQCT with gold standard micro-CT (R^2^ = 0.97) (Burghardt et al., 2007). Conversely, compared to gold standard micro-CT segmentation, the ARG segmentation method had a lower agreement than the manufacturer's default segmentation method (R^2^ = 0.86 vs R^2^ = 0.94) (Klintström et al., 2016).

**Fig. 4:**
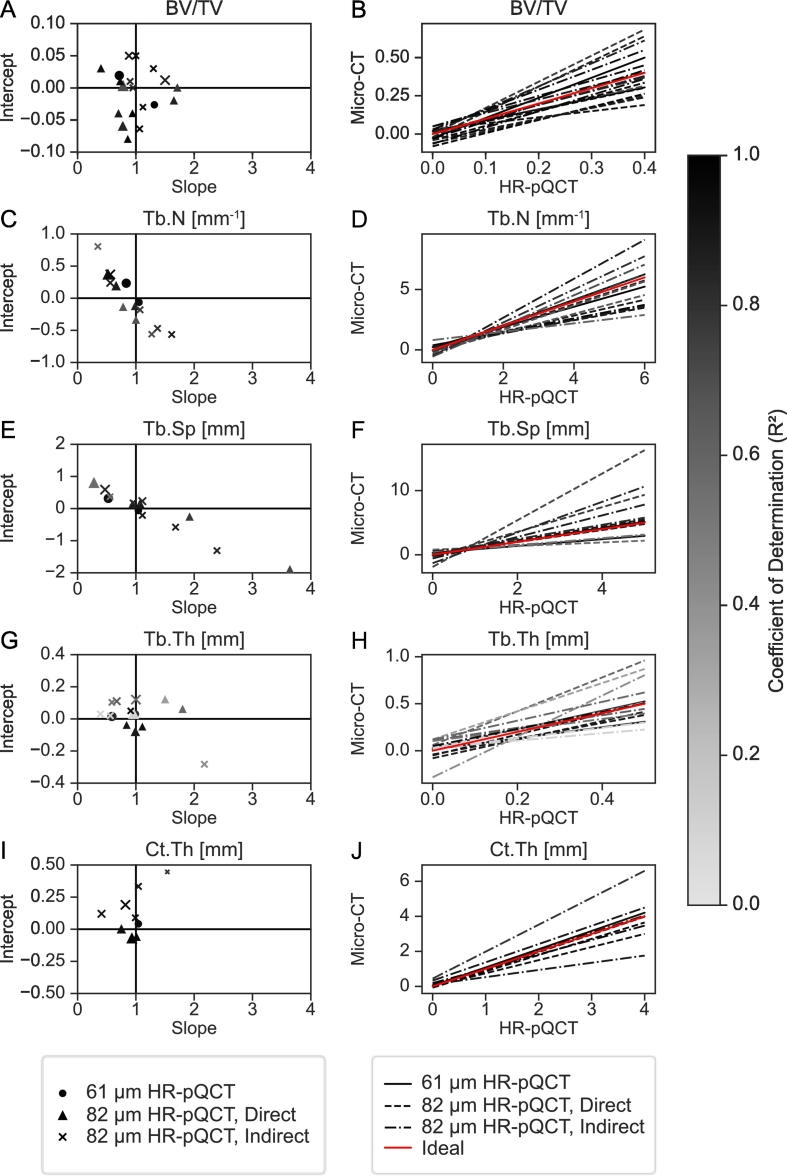
Linear regression analysis shows large variation across studies in the most commonly reported morphometric parameters, which were reported in more than ten studies. (A, C, E, G, I) Scatter plots of slope vs intercept for 61 μm HR-pQCT and 82 μm HR-pQCT validated against micro-CT show variability between parameters. Results from 82 μm HR-pQCT studies are further divided into direct and indirect approaches for all parameters. Grey-levels indicate the corresponding coefficient of determination (R^2^), while marker size indicates the voxel size of the micro-CT data used for comparison. (B, D, F, H, J) Linear regression lines from the same regression analyses are shown. The red, ideal line, indicates a perfect match between HR-pQCT and micro-CT and has slope of one and intercept of zero. In general, we observe that the parameters that have higher average coefficient of determination (e.g. BV/TV, Ct.Th) exhibit either under- or overestimation throughout the entire parameter space. In contrast, the parameter with the lowest coefficient of determination (e.g. Tb.Th) does not show a clear trend across studies. Furthermore, Tb.N and Tb.Sp have a consistent turning point between over- and underestimation across all studies.

Regression analysis between HR-pQCT and micro-CT BV/TV varied widely, with slopes ranging from 0.40 to 1.71 and intercepts ranging from −0.08 to 0.05 (Fig. 4A). There was a trend for underestimation of BV/TV for studies using the indirect method, while studies using the direct method showed a trend for overestimation (Fig. 4A, B). The two 61 μm HR-pQCT studies had a narrower range in slope and intercept than the 82 μm studies without clear over- or underestimation of BV/TV (Fig. 4A). Overall, due to the even spread of slope and intercept values, no standardized calibration for BV/TV could be identified.

#### 4.2.2 Trabecular number

Trabecular number (Tb.N) agreed well with the values generated from micro-CT for 82 μm and 61 μm HR-pQCT (R^2^ = 0.83, 0.94) (Fig. 3). The strength of this agreement for 82 μm devices was affected by disease state and anatomical location. Lower agreement (R^2^ = 0.67 to 0.81, depending on the computational method used) was observed for osteoporotic bone samples, which was attributed to an increased number of thin trabeculae that could not be assessed accurately with 82 μm HR-pQCT (Krause et al., 2014). An even lower agreement between HR-pQCT and micro-CT derived Tb.N (R^2^ = 0.61) was observed in the calcaneus (Metcalf et al., 2018). The local adaptive threshold strategy improved direct agreements with gold standard micro-CT Tb.N for 82 μm HR-pQCT compared to the indirect manufacturer's approach (R^2^ = 0.85 vs R^2^ = 0.70) for femoral head samples (Burghardt et al., 2007). While the manufacturer's approach relies on estimated parameters such as an assumed compact tissue density (Laib and Rüegsegger, 1999b), which might be patient specific and disease dependent, similar levels of agreement have been reported for radii using the manufacturer's indirect approach (R^2^ = 0.82) (MacNeil and Boyd, 2007). Relative to gold standard micro-CT, the ARG segmentation based direct method showed lower agreement than the manufacturer's default approach for the radius (R^2^ = 0.66 vs R^2^ = 0.81) (Klintström et al., 2016). In total, these studies suggest that the selection of an appropriate segmentation approach may be heavily dependent on the studied anatomical site.

Regression analysis performed by several studies showed a range of slopes of 0.35 to 1.61 with intercepts ranging from −0.56 to 0.81 mm^−1^ (Fig. 4C). Most studies showed a value-dependent over- and underestimation with the turning point being slightly above 1 mm^−1^ (Fig. 4D). No clear agreement was observed for slope and intercept between the different studies, however, reduced variability was observed for the 61 μm compared to 82 μm studies (Fig. 4C).

#### 4.2.3 Trabecular separation

Agreements similar to those reported for Tb.N were found for trabecular separation (Tb.Sp) (R^2^ = 0.87, 0.94) (Fig. 3). The range of agreement in Tb.Sp was also similar to that of Tb.N (Fig. 3), as is expected since they are reciprocals. Interestingly, the trapezium study found Tb.Sp values from 82 μm images agreed better with micro-CT than Tb.Sp values from 61 μm images (R^2^ = 0.93 vs R^2^ = 0.88) (Mys et al., 2019). Some studies found better agreement for the indirect, manufacturer recommended method (Krause et al., 2014; MacNeil and Boyd, 2007), which is based on grey-scale BV/TV and indirect Tb.N (Laib et al., 1998). Others found no difference between Tb.Sp measured using the indirect and direct segmentation based methods (Liu et al., 2010; Zhou et al., 2016). The direct local adaptive threshold method resulted in higher agreement than the indirect method (R^2^ = 0.93 vs R^2^ = 0.85) (Burghardt et al., 2007). One of the studies reporting higher agreement for indirect methods evaluated osteoporotic samples (Krause et al., 2014), however no osteoporotic samples were analysed by Burghardt and colleagues. Therefore, it is unclear whether the observed differences between direct and indirect methods are due to the segmentation method or the bone quality of the samples. The direct ARG segmentation method resulted in lower agreement than the indirect method (R^2^ = 0.52 vs R^2^ = 0.64) (Klintström et al., 2016) and is not recommended for computing Tb.Sp. Overall, the accuracy with which Tb.Sp can be assessed and the preferred method of calculation seems dependent on the size of the trabeculae relative to the voxel size.

The regression results from all studies resulted in slopes in the range of 0.28 to 3.64 with intercepts from −1.90 to 0.80 mm (Fig. 4E). As with Tb.N, a turning point was observed for Tb.Sp slightly below 1 mm, which is roughly the inverse of the turning point for Tb.N (Fig. 4F). Overall, no clear agreement was observable for the regression parameters, while reduced ranges for the regression parameters were observed for 61 μm images compared to 82 μm images (Fig. 4E).

Interestingly, Tb.Sp was inconsistently defined as either trabecular spacing or trabecular separation. By definition, trabecular spacing refers to the distance between midlines of trabeculae, while trabecular separation describes the distance between edges of trabeculae. The source data for Tb.Sp, whether defined as separation or spacing, has not been modified herein.

#### 4.2.4 Trabecular thickness

For the commonly reported trabecular metrics, trabecular thickness (Tb.Th) performed the poorest (R^2^ = 0.59, 0.82) (Fig. 3). The direct measure of Tb.Th for 82 μm HR-pQCT images, either using fixed or local adaptive threshold segmentation, agreed better than or equally as well as the indirect method of the manufacturer (R^2^ = 0.80 vs R^2^ = 0.55), which is based on grey-scale BV/TV and indirect Tb.N (Laib et al., 1998). As an exception, one study reported better performance of the manufacturer's indirect method (MacNeil and Boyd, 2007). While they argued that the poor agreement found for the direct method from 82 μm HR-pQCT (R^2^ = 0.08) could be a result of the inherent low resolution of the images, this did not seem to hinder the other studies assessed herein. This apparent contradiction may be a result of variation across studies in pre-processing of the image data for the direct method. Hence, future studies looking at Tb.Th should also look at the sensitivity to different pre-processing protocols.

Slopes ranged from 0.39 to 2.17 and intercepts ranged from −0.28 to 0.12 mm (Fig. 4G). Since the indirect Tb.Th is a derived parameter, often represented as Tb.Th* or Tb.Th^d^, dependent on grey-scale BV/TV and indirect Tb.N, which showed no clear trend in terms of over- or underestimation, the linear regressions found for indirect Tb.Th also did not show a clear over- or underestimation trend (Fig. 4H). As with other parameters, 61 μm images showed reduced variability compared to the 82 μm images (Fig. 4H).

#### 4.2.5 Bone surface to bone volume ratio

Bone surface to bone volume ratio (BS/BV) was only included in three studies (Liu et al., 2010; MacNeil and Boyd, 2007; Zhou et al., 2016), all of which used 82 μm HR-pQCT and scans of radii or tibiae. Even though, all studies used the same method to compute BS/BV (Müller et al., 1994), two studies found good agreement (R^2^ = 0.83) (Liu et al., 2010; Zhou et al., 2016) and one study found poor agreement (R^2^ = 0.18) with BS/BV measured using micro-CT (MacNeil and Boyd, 2007). No alternative segmentation method was tested nor were any distinct patient populations included in these studies.

#### 4.2.6 Structural model index

Structural model index (SMI) provides a metric for how plate- or rod-like a trabecular architecture is. However, one study cautioned against this interpretation of SMI, as a loss in BV/TV has been shown to shift SMI to indicate a more rod-like bone structure (Salmon et al., 2015). All five studies reporting SMI utilized 82 μm HR-pQCT images. The same study that found poor agreement for BS/BV found poor agreement for SMI (R^2^ = 0.08) (MacNeil and Boyd, 2007). However, the other studies found moderate agreement (R^2^ = 0.78) (Burghardt et al., 2007; Krause et al., 2014; Liu et al., 2010; Zhou et al., 2016) with the highest agreement found for the local adaptive threshold (R^2^ = 0.92) (Burghardt et al., 2007). Since the study with osteoporotic bone samples found lower agreement between SMI from micro-CT and 82 μm HR-pQCT data (Krause et al., 2014), the accuracy for SMI may depend on the disease status or bone quality of the scanned patient. Although the local adaptive threshold procedure produced results most similar to micro-CT SMI, this pre-processing method was only tested on non-osteoporotic bone samples. Further validation should be performed before this method can be recommended unconditionally as a pre-processing step for the computation of SMI.

#### 4.2.7 Connectivity density and the degree of anisotropy

Connectivity density (Conn.D) is a computational measure of the inter-connectivity among trabeculae and can be an indicator of the mechanical strength of the trabecular architecture (Odgaard and Gundersen, 1993). Degree of anisotropy (DA) is a measure that describes the degree to which trabeculae are oriented along a common axis (Harrigan and Mann, 1985). For Conn.D and DA only slight and moderate agreement was found for 82 μm HR-pQCT, respectively (R^2^ = 0.50 for Conn.D, R^2^ = 0.62 for DA) (Burghardt et al., 2007; Krause et al., 2014; Liu et al., 2010; MacNeil and Boyd, 2007; Zhou et al., 2016). No comparisons between 61 μm HR-pQCT and micro-CT have been performed for Conn.D and DA.

#### 4.2.8 Less common parameters

Trabecular nodes (Tb.Nd) is the count of trabecular intersections. Trabecular termini (Tb.Tm) is the number of free ends in the trabecular structure. Only one study evaluated Tb.Nd and Tb.Tm and found slight to moderate agreement (R^2^ = 0.49 to 0.74) using the ARG segmentation algorithm for 82 μm HR-pQCT (Klintström et al., 2016). The standard manufacturer's segmentation resulted in poor agreement for these parameters (R^2^ = 0.07).

Mean intercept length (MIL), a measure of micro-architectural anisotropy (fabric), agreed well between 82 μm HR-pQCT and micro-CT, even when different filtering techniques for pre-processing were applied (R^2^ = 0.97 vs R^2^ = 0.99 for Laplace Hamming and Gaussian filtering, respectively) (Varga and Zysset, 2009). Using 82 μm HR-pQCT, Hosseini and colleagues showed that the manufacturer's function to evaluate DA (TRI (Laib et al., 2000)) and their own open source implementation, mean surface length (MSL), both agreed well with MIL computed on micro-CT (R^2^ > 0.98 for all) (Hosseini et al., 2017). Here, the same threshold (320 mg HA/cm^3^) was used for micro-CT and HR-pQCT, demonstrating that fabric allows for more direct comparison between the two imaging modalities. However, while other studies looking at DA assessed the entire trabecular compartment, Hosseini and colleagues used three hand-selected cubic regions of interest (ROIs) (edge length 6 mm) per sample. This ROI-dependent precision (R^2^ = 0.99 vs R^2^ = 0.62) shows the importance of standardizing ROI selection to ensure cross-study comparability.

Individual trabecula segmentation (ITS) is another approach, implemented in commercial software, to extract morphometric information from a CT image that differentiates between plate- and rod-like structures (Liu et al., 2011). Agreement between plate parameters was generally higher than for rod parameters. The declared conflict of interest for ITS by Liu and colleagues calls for independent research groups to substantiate these results.

#### 4.2.9 Effects of segmentation on trabecular parameters

The effect of custom segmentation methods on trabecular parameters remains unclear. The local adaptive threshold segmentation method performed equivalently or better for the analysis of morphological parameters of trabecular bone, e.g. Tb.N, Tb.Sp, etc., when compared to the global threshold segmentation method (Burghardt et al., 2007). Similarly, MSL was extracted accurately with a fixed threshold segmentation (Hosseini et al., 2017). However, others observed that the scanner manufacturer's compartmental segmentation and indirect grey-scale method yielded better estimates of BV/TV and most morphometric indices, respectively, compared to micro-CT based or their proposed segmentation approaches (Klintström et al., 2016; Varga and Zysset, 2009). Simultaneously, their alternative segmentation approaches outperformed the manufacturer's approach when assessing less common parameters, such as Tb.Tm or MIL.

### 4.3 Cortical parameters

Due to the low resolution of HR-pQCT images and their increased noise, segmenting the cortex accurately, especially identifying pores and the correct endosteal surface, can be challenging. Consequently, parameters derived from these segmentations require a thorough validation against a high-quality gold standard such as micro-CT.

#### 4.3.1 Cortical thickness

Agreement between micro-CT and HR-pQCT measured cortical thickness (Ct.Th), the most commonly reported cortical parameter, was higher than most other cortical parameters for both device resolutions (R^2^ = 0.92, 0.94) (Liu et al., 2010; MacNeil and Boyd, 2007; Nishiyama et al., 2010; Ostertag et al., 2016; Tjong et al., 2012; Zhou et al., 2016) (Fig. 3). The dual threshold segmentation algorithm improved agreement in Ct.Th for 82 μm images in two studies compared to Ct.Th derived from images using the indirect method in the scanner manufacturer's software. In one study, both had good agreement (R^2^ = 0.98 vs R^2^ = 0.90) (Nishiyama et al., 2010), while in the other study, good agreement was observed in directly computed Ct.Th derived from dual threshold segmented images and only moderate agreement in Ct.Th derived from images using the indirect method in the scanner manufacturer's software (R^2^ = 0.85 vs R^2^ = 0.79) (Ostertag et al., 2016). The manufacturer's indirect approach for Ct.Th uses an assumed relation of cortical volume over outer bone surface (MacNeil and Boyd, 2007), which could explain the lower agreement observed for the indirect compared to the direct method.

Regression analyses found that Ct.Th computed from HR-pQCT matched Ct.Th from gold standard micro-CT much better if the direct segmentation-based method was used (Fig. 4I, J). Slopes ranged from 0.41 to 1.54 and intercepts ranged from −0.07 to 0.45 mm (Fig. 4I). Only one 61 μm study assessed Ct.Th (Tjong et al., 2012) and reported a good match with micro-CT (Fig. 4I), but it should be noted that Tjong and colleagues used an 82 μm HR-pQCT device in non-patient mode with a resolution of 41 μm. Since this resolution is higher than the resolution of 61 μm HR-pQCT devices, further studies should clarify the effect this difference in resolution has on the accuracy of Ct.Th measured using 61 μm HR-pQCT.

#### 4.3.2 Cortical bone mineral density

One study reported slight agreement (R^2^ = 0.59) with cortical bone mineral density (Ct.BMD) for an 82 μm HR-pQCT device running in non-patient mode (41 μm resolution) and a weaker agreement for the normal 82 μm HR-pQCT resolution (R^2^ = 0.44) (Tjong et al., 2012). No direct comparison between a 61 μm HR-pQCT device and micro-CT has been performed thus far. Given that only one 82 μm HR-pQCT study has evaluated Ct.BMD relative to micro-CT, the accuracy with which HR-pQCT can measure this parameter remains unclear.

#### 4.3.3 Cortical porosity

Cortical porosity (Ct.Po) agreed well for both HR-pQCT resolutions (R^2^ = 0.85, 0.84) (Jorgenson et al., 2015; Nishiyama et al., 2010; Ostertag et al., 2014, Ostertag et al., 2016; Soltan et al., 2019; Tjong et al., 2012). For 82 μm HR-pQCT, the direct 3D evaluation yielded better agreement than the indirect density derived evaluation (R^2^ = 0.85 vs R^2^ = 0.75). Using StrAx1.0, Ct.Po measurements agreed well (R^2^ = 0.87 to 0.98) (Zebaze et al., 2013), while with the direct approach, the number of detected pores agreed poorly (R^2^ = 0.27) (Nishiyama et al., 2010). This discrepancy could be due to the inability of HR-pQCT to detect the smallest pores, thus reducing the pore count while not greatly affecting the measured porosity.

#### 4.3.4 Less common parameters

StrAx1.0 assesses cortical area using three parameters, total cross-sectional area, the compact-appearing cortex area, and the transitional zone area (Fig. 1C). These parameters, measured using 82 μm HR-pQCT data, agreed well with their micro-CT measured equivalents (R^2^ = 0.99, 0.98, 0.95, respectively). However, agreement with the scanner manufacturer's software was poor (R^2^ = 0.32) (Zebaze et al., 2013).

The parameter of matrix mineral density (MMD) was introduced to address that Ct.BMD does not differentiate between a reduced mineralized bone matrix volume and reduced mineralisation of the matrix. Moderate agreement was found between MMD computed with StrAx1.0 and with the manufacturer's software for micro-CT data (R^2^ = 0.76) (Chiang et al., 2018).

While cortical interruptions can be used as a predictor of progressing rheumatoid arthritis (Scott, 2003), only moderate inter-rater reliability was found for the visual detection of cortical interruptions with diameters of less than 0.5 mm using 82 μm HR-pQCT and micro-CT, despite the extensive training and prior experience of the operators (Scharmga et al., 2016). To avoid inter-rater variability, an algorithm was proposed for detecting cortical interruptions (Peters et al., 2017), which modified the default segmentation of the manufacturer. This algorithm identified similar cortical interruptions for both HR-pQCT and micro-CT images and performed best for interruptions with a diameter of at least 0.16 mm for 82 μm HR-pQCT (inter-class correlation coefficient = 0.91).

#### 4.3.5 Effects of segmentation on cortical parameters

Only a few studies have looked at the effects of segmentation on cortical parameters. The thickness-based separation segmentation approach achieved sub-voxel precision on some of the cortical morphometric parameters (Ang et al., 2020), while the use of manually-corrected segmentations resulted in inter-observer reproducibility errors of 3.7% for Ct.Th, 5.3% for Ct.BMD, and 6.2% for cortical porosity (Ostertag et al., 2014). Further studies should investigate the replacement of manual hand-drawn contours and manually-corrected segmentations with automatic approaches to eliminate such uncertainties and enable more direct cross-study and cross-centre comparisons.

### 4.4 Discussion

Few 61 μm HR-pQCT studies have validated morphometrics against micro-CT and the accuracy of 61 μm HR-pQCT cannot be assessed from comparisons of 82 μm and 61 μm HR-pQCT without a proper gold standard (Agarwal et al., 2016; Manske et al., 2015, Manske et al., 2017), especially for parameters which do not agree well between 82 μm HR-pQCT and micro-CT. Importantly, a clear difference in morphological parameter agreement relative to analysis type was found between 82 and 41 μm HR-pQCT (Tjong et al., 2012); here, indirect methods resulted in a better agreement with the parameters derived from micro-CT for 82 μm HR-pQCT, while direct methods resulted in better agreement for 41 μm HR-pQCT. Given that 61 μm HR-pQCT is between these two resolutions, it is not obvious which method best analyses 61 μm HR-pQCT images.

There are a number of issues which may hinder the development of a generalized validation of HR-pQCT relative to micro-CT. Perhaps the largest issue with the comparison is the lack of a standardized approach for pre-processing micro-CT data. The plethora of pre-processing steps described for micro-CT might explain the systematic deviations between studies, which are clearly visible in the regression results for all morphometric parameters (Fig. 4).

Micro-CT resolution was also not standardized across studies, which is especially relevant for trabecular indices, such as Tb.Th, which have a strong dependence on image resolution (Kim et al., 2004; Kothari et al., 1998; Müller et al., 1996; Sode et al., 2008). Müller reported that resolutions of less than 10 μm should be used to obtain accurate results (Müller, 2003). Yet, we observed a wide variety of micro-CT resolutions, with some exceeding 30 μm (Table 2). Since the accuracy of HR-pQCT is not expected to match that of micro-CT, the improved accuracy of using <10 μm micro-CT for comparison is unlikely to affect the validation of HR-pQCT. Importantly, we did not observe dependence on scanner resolution for morphologic parameters (Fig. 3). However, studies looking at very thin, i.e. osteoporotic, micro-architectures used resolutions below 20 μm for validation. The low resolutions of HR-pQCT devices may require the use of various segmentation methods to yield accurate results for all morphological parameters (Varga and Zysset, 2009).

The accuracy for trabecular parameters varied by anatomical site, with the calcaneus having less accurate results with HR-pQCT compared to the radius or the tibia. This discrepancy may be a result of the morphometric analysis methods being fine-tuned for the distal radius and tibia, necessitating the development of a more universal or site-specific evaluation function.

Different implementations have been introduced to compute the various morphometric indices; unfortunately, only the groups that proposed these implementations have reported on their accuracy. The lack of validation by independent research groups limits the amount of data, relative to anatomical site, disease status, etc., that is available for cross-comparison. This lack of comparative data, especially when existing datasets are overly homogenous, can lead to conflicts of interest when only internally-produced data is used for the validation of proprietary algorithms or derived commercial products. The introduction and use of a publicly available database of diverse validation data could help to avoid such issues in the future.

Finally, only static cortical and trabecular parameters have been evaluated; dynamic measures for HR-pQCT, such as bone formation and resorption parameters, have yet to be investigated (Schulte et al., 2011). Since longitudinal micro-CT data cannot be generated for patients, other validation methods are required to assess these dynamic parameters. In silico models can now provide both realistic bone structures and simulated time-lapse data. By coupling these models with simulated image artefacts, such as noise, a wide variety of input data could be generated to evaluate dynamic parameters that extend what is possible with a single micro-CT or HR-pQCT scan.

## 5 Finite element analysis (FEA)

Shortly after the introduction of micro-CT, researchers began investigating the use of imaging to not only quantify bone structure but also evaluate bone strength through FEA (Rüegsegger et al., 1996; van Rietbergen et al., 1995). Importantly, non-destructive micro-CT FEA methods have since been validated against experimentally derived measures of strength and failure (Chen et al., 2017; Hambli, 2013). With the introduction of HR-pQCT for patient imaging in the clinic, it became evident that measures of strength and failure prediction from HR-pQCT FEA may aid in patient diagnosis and treatment. However, the application of FEA methods to lower resolution images requires thorough validation before these benefits can be realized.

To date, 11 studies have utilized FEA in their analysis of 82 μm HR-pQCT relative to micro-CT; 61 μm HR-pQCT FEA has yet to be evaluated relative to micro-CT FEA. Of these 11 studies, eight included a comparison of mechanical measures between HR-pQCT FEA and micro-CT FEA, three of which also included comparisons of mechanical measures to experimentally derived measures, three utilized micro-CT FEA to evaluate the ability of HR-pQCT image morphometrics to predict mechanical parameters, and one varied the voxel size of the input micro-CT data to isolate its effect on the FEA (Table 3). Seven studies used hexahedral elements and an isotropic linear-elastic material model, three used hexahedral elements and an elastic-plastic model with 5% or 50% reduction in elastic modulus after 0.33% tensile strain or 0.81% compressive strain, and one study also used quadratic pentahedral and tetrahedral elements in a homogenized FEA with an orthotropic elasticity tensor (Table 3). Similar to the Section 4, data from previous studies is presented as *slope* * value_HR-pQCT_ + *intercept* = value_micro-CT_. Due to the lack of consistent reporting, all R^2^ values are specific to a single study.

**Table 3:** Parameters of HR-pQCT and micro-CT finite element analyses used to validate the use of HR-pQCT.

Study	Material model	Element type (voxel size)	Micro-CT geometry^a^	HR-pQCT geometry^a^	Loading conditions	FE solver	Study measures	Purpose
(MacNeil and Boyd, 2007)	Isotropic, linear elastic (E = 10 GPa, ν = 0.3)	Hexahedral (19 μm/82 μm)	164x164x164 (radius, 3.12 mm)	38x38x38 (radius, 3.12 mm)	1.0% Comp	Custom (Su et al., 2007)	Reaction force, stress, SED	Validation (micro-CT)
(Liu et al., 2010)	Isotropic, linear elastic (E = 15 GPa, ν = 0.3)	Hexahedral (40 μm/82 μm)	143x143x143 (tibia, 5.72 mm) 10 mm CS (tibia)	70x70x70 (tibia, 5.74 mm) 9.02 mm CS (tibia)	SV: Combi; CS: 1.0% Comp (van Rietbergen, 1996)	Olympus (Adams, 2002; Adams et al., 2004; Laib, 1997)	SV: elastic and shear moduli; CS: trabecular and total stiffness	Validation (micro-CT)
(Alsayednoor et al., 2018)	Isotropic, linear elastic^b^ (E = 10 GPa, ν = 0.33)	Hexahedral (17.41 μm/82 μm)	301x301x301 (calcaneus, 5.24 mm)	65x65x65^c^ (calcaneus, 5.33 mm)	0.13% Comp	Abaqus	Reaction force, von Mises stress, failure load	Validation (micro-CT)
(Cohen et al., 2010)	Isotropic, linear elastic (E = 15 GPa, ν = 0.3)	Hexahedral (8 μm/82 μm)	640x640x300 (iliac crest, 2.40 mm)	70x70x70 (radius, 5.74 mm) 110x110x110 (tibia, 9.02 mm)	Combi (Hollister et al., 1994; van Rietbergen, 1996)	Custom (Arbenz et al., 2008)	Elastic Moduli	Comparison (micro-CT)
(Liu et al., 2013)	Isotropic, elastic-plastic (E_HR-pQCT_ = 16.59 (5%) GPa, E_μCT_ = 10.43 (5%) GPa, ν = 0.3)	Hexahedral (25 μm/82 μm)	230x230x230 (radius/tibia, 5.75 mm)	70x70x70 (radius, 5.74 mm) 110x110x110 (tibia, 9.02 mm)	1.0% Comp^a^	Olympus (Adams et al., 2004)	Elastic moduli, yield strength	Validation (micro-CT), fracture risk prediction
	(E = 39.62 (5%) GPa, ν = 0.3)	PR (--/82 μm)	–	9.02 mm CS (radius/tibia)	1.0% Comp^a^	Abaqus		
(Zhou et al., 2016)	Isotropic, elastic-plastic (E = 15 (50%) GPa, ν = 0.3)	Hexahedral (37 μm/82 μm)	Approx. 9 mm CS (radius/tibia)	9.02 mm CS (radius/tibia)	1.2% Comp	FEAP (Adams et al., 2004)	Total stiffness, yield strength, morph	Validation (Exp, micro-CT)
(Wang et al., 2019)	Isotropic, elastic-plastic (E = 15 (50%) GPa, G = 7 GPa^e^, ν = 0.3)	Hexahedral (37 μm/82 μm)	9.02 mm CS (radius/tibia)	9.02 mm CS (radius/tibia)	1.2% Comp	FEAP (Adams et al., 2004)	Total stiffness, total yield strength	Validation (Exp, micro-CT), fracture risk prediction
		PR with hexahedral cortex (--/82 μm)	–	9.02 mm CS (radius/tibia)	1.2% Comp	Abaqus		
(Pahr et al., 2012)	Isotropic, linear elastic^b^ (E_BMD_ = 21.96(BV/TV)^1.7^ GPa or E_SEG_ = 8.78 GPa, ν = 0.3^b^)	Hexahedral (--/82 μm)	–	19.33 ± 2.15 mm CS (Vertebra)^b^	Exp, Comp (Dall'Ara et al., 2010)	ParFE (Arbenz et al., 2008)	Elastic modulus	Validation (Exp)
(Pahr et al., 2012)	Homogenized, orthotropic elastic (E = 12 GPa, G = 3.913 GPa, ν = 0.249)	Pentahedral cortex/tetrahedral trabecular with fabric (--/82 μm)	–	19.33 ± 2.15 mm CS (vertebra)^b^	1.2% Comp	Abaqus	Orthotropic stiffness	Validation (Exp)
(Christen et al., 2016)	Isotropic, linear elastic^b^ (E = 6.8 GPa, ν = 0.3)	Hexahedral (25 μm/--)^d^	9.02 mm CS (radius)	–	Unit Comp (3 uniaxial directions)	ParOSol (Flaig, 2012)	Load estimate error	Voxel size dependency
(Liu et al., 2011)	Isotropic, linear elastic^b^ (E = 15 GPa, ν = 0.3)	Hexahedral (40 μm/--)	143x143x143 (tibia, 5.72 mm)	–	Combi (van Rietbergen, 1996)	FEAP (Adams et al., 2004)	Elastic modulus, morph	Micro-FEA evaluation of morph
(Klintström et al., 2016)	Isotropic, linear elastic^b^ (E = 12 GPa, ν = 0.3)	Hexahedral (20 μm/--)	260x260x260 (radius, 5.20 mm)	–	Combi (Chevalier et al., 2008)	Abaqus	Stiffness, shear moduli, morph	Micro-FEA evaluation of morph

Other terms: SV, sub-volume of bone isolated from the trabecular compartment; PR, plate-rod geometry consisting of 2-node rods and 3-node shell plates; CS, bone cross-section; Combi, a combination of six micro-FEA analyses including three uniaxial compressions and three uniaxial shear; Comp, uniaxial compression along the long axis of the bone; Exp, experimental; FEAP, Finite Element Analysis Program; SED, strain energy density; Morph, morphometrics derived from HR-pQCT images.

a Subvolume sample geometry is presented as voxel dimensions (bone source, subvolume height) and bone cross-section sample geometry is presented as cross-section height (bone source).

b Assumed value, not available in text.

c Data was evaluated using three methods: resampling to micro-CT resolution, thresholded to match BV/TV of micro-CT images, and thresholded to match fractal structure of micro-CT images.

d Data was resampled to 50, 61, 75, 82, 100, 125, and 150 μm for analysis at multiple voxel sizes.

e Shear modulus, G, only used in PR model.

### 5.1 Bulk mechanical properties

In studies comparing the mechanical response of FEA models from HR-pQCT to those from micro-CT, increased bone strength and stiffness estimations were observed with increasing voxel size (Alsayednoor et al., 2018; Cohen et al., 2010; Liu et al., 2010, Liu et al., 2011, Liu et al., 2013; MacNeil and Boyd, 2007; Wang et al., 2019; Zhou et al., 2019). Specifically, one study found reaction force was overestimated by HR-pQCT (y = −5 + 0.42x N; R^2^ = 0.73) (MacNeil and Boyd, 2007). However, another study found that both reaction force and failure load estimates depend on the chosen threshold, such that a BV/TV-matched threshold led to significant underestimations, while a geometry-preserving threshold led to general overestimations (Alsayednoor et al., 2018). Due to the small sample size (*N* = 5), no regressions were performed, and the variability between specimens was notably large (Alsayednoor et al., 2018). Regarding analysis type, strength was underestimated using linear HR-pQCT FEA for the radius (y = −0.92 + 1.7x kN; R^2^ = 0.92) and for the tibia (y = −0.39 + 1.5x kN; R^2^ = 0.96) and overestimated using nonlinear HR-pQCT FEA for the radius (y = −0.31 + 0.84x kN; R^2^ = 0.91) and for the tibia (y = 0.77 + 0.76x kN; R^2^ = 0.95) (Zhou et al., 2016).

Stiffness was also overestimated through HR-pQCT FEA. This was observed in the evaluation of trabecular bone stiffness (y = −16.6 + 0.69x kN/mm; R^2^ = 0.90 (Liu et al., 2010)) and whole bone stiffness (y = −24.6 + 0.87x kN/mm; R^2^ = 0.96 (Liu et al., 2010) and y = 4.3 + 0.86x kN/mm; R^2^ = 0.94 (Zhou et al., 2016)) for the tibia. Similar results were found for stiffness of the radius (Zhou et al., 2016), but are not reported herein. While the slopes for whole bone stiffness of the tibia were similar between the two studies, the intercept varied in both sign and magnitude, possibly due to the use of different material models (linear elastic vs elastic-plastic, respectively).

With respect to tissue-level properties, strain energy density was overestimated by HR-pQCT (y = 0.004 + 0.42x J/mm^3^; R^2^ = 0.50) (MacNeil and Boyd, 2007). Two studies presented conflicting results in their comparison of average von Mises stress between micro-CT and HR-pQCT FEA. In one study, von Mises stress was overestimated by HR-pQCT (y = 2.5 + 0.46x MPa; R^2^ = 0.51) (MacNeil and Boyd, 2007), while another study found von Mises stress from HR-pQCT-based models was either equivalent or underestimated depending on the threshold used; however, no regression or quantification was performed in this second study (Alsayednoor et al., 2018).

The study which explicitly evaluated voxel size using micro-CT images found increased error in estimated loading with increased voxel size (Christen et al., 2016). Similarly, the use of a BV/TV-matched threshold on downsampled micro-CT images resulted in slight underestimations of failure load and significant underestimations of apparent stiffness and von Mises stresses in comparison to native resolution (Alsayednoor et al., 2018). In contrast, a third study noted that continuum parameters, e.g. reaction force and von Mises stress, correlated better with results from micro-CT FEA than tissue-level parameters, e.g. strain energy density (MacNeil and Boyd, 2007). The inconsistencies indicate that further research is warranted to elucidate the true effect of both voxel size and imaging modality on FEA-derived mechanical properties.

### 5.2 Tissue mechanical properties

Several studies utilized both HR-pQCT and micro-CT FEA to assess isotropic or orthotropic elastic moduli of a cubic trabecular bone section; herein, elastic moduli were overestimated when HR-pQCT images were utilized (Cohen et al., 2010; Liu et al., 2010, Liu et al., 2013). This evaluation method was first used to compare images acquired from different anatomical locations and found HR-pQCT-derived apparent Young's moduli to be poor predictors (R^2^ < 0.19) of micro-CT values (Cohen et al., 2010). Specifically, when compared to micro-CT values from the iliac crest, mean apparent Young's moduli in the longitudinal direction was overestimated by HR-pQCT by an average of 46% (305 MPa) and 54% (358 MPa) for the tibia and radius, respectively. Due to the use of varied anatomical sites, including both weight-bearing and non-weight-bearing samples, it was difficult to draw conclusions about the specific effect of using micro-CT vs HR-pQCT (Cohen et al., 2010). The second study to evaluate the apparent elastic moduli from HR-pQCT and micro-CT FEA found an overestimation of elastic moduli (longitudinal, y = −403 + 0.83x MPa; R^2^ = 0.92), but also measured average BV/TV to be 0.25 for HR-pQCT and 0.14 for micro-CT which may help to explain these differences (Liu et al., 2010).

The use of varied material models and properties for micro-CT and HR-pQCT FEA models eliminated significant differences in apparent elastic moduli (relationship not quantified) (Liu et al., 2013; Pahr et al., 2012). For one such study, material properties derived on a separate cadaveric cohort were applied to a cohort of 60 patients, enabling the differentiation between patients with and without previous vertebral fractures (Liu et al., 2013). Interestingly, experimentally derived apparent stiffness was relatively well predicted using both the voxel-based BMD (y = −0.02 + 1.03x GPa, R^2^ = 0.86) and binarised (y = 0.07 + 0.88x GPa, R^2^ = 0.84) FEA models (Pahr et al., 2012).

### 5.3 Non-voxel-based FEA

In an effort to reduce the computational time associated with micro-FEA, two alternative methods were introduced for HR-pQCT FEA and validated against micro-CT FEA and experimental findings (Liu et al., 2013; Pahr et al., 2012; Wang et al., 2019). The first of these methods included a fabric-based homogenization method which was applied to vertebral samples, calibrated using micro-CT FEA, and evaluated against experimental measures (Pahr et al., 2012). This method was more computationally efficient and was able to predict apparent stiffness relatively well in comparison to experimental measures using BMD-based (y = 0.06 + 1.01x GPa, R^2^ = 0.75) and binarised (y = −0.04 + 1.07x GPa, R^2^ = 0.86) model inputs (Pahr et al., 2012). The second method was a result of a geometry simplification process, i.e. ITS, which resulted in a plate-rod (PR) geometry consisting of 2-node rods and 3-node shell plates (Liu et al., 2011). PR FEA resulted in similar predictions of elastic modulus (y = 2.42 + 0.79x MPa; R^2^ = 0.91) and yield strength after initial adjustment of material properties (y = 2.36 + 0.72x MPa; R^2^ = 0.86) relative to voxel-based micro-CT FEA (Liu et al., 2013). A follow-on study utilized this method in the evaluation of stiffness and yield strength predictions against those from both micro-CT FEA and mechanical testing (Wang et al., 2019). Here, stiffness from HR-pQCT PR FEA was a good estimate for voxel-based micro-CT FEA (y = −6.63 + 1.04x kN/mm; R^2^ = 0.94) and values from mechanical testing (y = −26.5 + 1.06x kN/mm; R^2^ = 0.88) in a pooled dataset from the radius and tibia. Yield strength from HR-pQCT PR FEA was also a good estimate of voxel-based micro-CT FEA (y = −1.38 + 1.16x kN; R^2^ = 0.95), but overestimated yield strength (y = −1.17 + 1.30x kN; R^2^ = 0.94) for the same pooled dataset.

### 5.4 Morphological assessments

BV/TV was found to be an independent predictor of whole bone stiffness and apparent elastic moduli (R^2^ = 0.49 to 0.74 for HR-pQCT and R^2^ = 0.65 to 0.82 for micro-CT) (Liu et al., 2010). Interestingly, studies evaluating HR-pQCT morphometrics relative to micro-CT FEA also found strong agreement of micro-CT FEA mechanical parameters with both BV/TV and Tb.Tm (Klintström et al., 2016); with total bone area (Tt.Ar), BMD, Ct.Th, and Tb.Th (Zhou et al., 2016); and with ITS-based metrics of trabecular plates, orientation, and structure (Liu et al., 2011). Therefore, while BV/TV has an inherent influence on the mechanical results from FEA due to its characterization of the anatomy, several other morphologic parameters appear to also be relevant to the prediction of mechanical properties for HR-pQCT imaged anatomy.

### 5.5 Discussion

Generally, the use of material models and material properties has been inconsistent across HR-pQCT FEA investigations. This holds true even for the gold standard of micro-CT FEA. Additionally, the mechanical properties and metrics measured by various study groups is inconsistent. Combined, these factors resulted in difficulty in categorizing and comparing results between studies and investigating trends in observed differences. Specifically, the most commonly reported parameter across studies was orthotropic elastic moduli, however these values have only been reported in three studies by the same research group, including one study which compared different anatomical sites.

HR-pQCT FEA models clearly tend to overestimate bone strength and elastic modulus when applied without additional calibration or material property adjustments. While the results from 61 μm HR-pQCT FEA have yet to be compared to micro-CT FEA, the overestimation of reaction force and failure load observed with 82 μm HR-pQCT FEA seems to be reduced when directly comparing 61 μm to 82 μm HR-pQCT FEA (Whittier et al., 2018). This difference in mechanical properties may be resultant of inherent differences in BV/TV and trabecular structure preservation between the two imaging modalities (Liu et al., 2010). However, one study investigated the effect of using either BV/TV-matched or geometry-preserving thresholds and found that neither method resulted in similar values to those obtained from micro-CT FEA for any of the mechanical measures evaluated (Alsayednoor et al., 2018). Despite this, mechanical properties from HR-pQCT FEA are still highly correlated with those from both micro-CT FEA and experimental findings, indicating that a correction may be possible.

Given the current state of the literature, HR-pQCT FEA may not be a feasible solution for accurately quantifying mechanical properties, but instead an excellent option for use in comparative studies. Accordingly, two studies have shown that HR-pQCT FEA models were able to distinguish between patients with and without previous radius fractures, indicating a potential clinical application of these models in the future (Liu et al., 2013; Wang et al., 2019). While comparative studies are sufficient for many clinical applications, the availability of diverse datasets, which include experimentally derived mechanical properties and both micro-CT and HR-pQCT images, would provide the necessary basis for future validation of the quantification of mechanical properties using HR-pQCT, leading to patient-specific applications of HR-pQCT FEA.

## 6 Discussion

The aim of this review was to provide an overview of the current status of computational method validation for HR-pQCT with respect to gold standard micro-CT and of the limited standardization across the field. We found that there is good agreement between HR-pQCT and micro-CT for a variety of morphometric or mechanical parameters. Notably, of the most commonly used morphometric parameters, BV/TV and Ct.Th had the highest agreement. While most segmentation methods are calibrated to yield accurate BV/TV values, Ct.Th is insensitive to partial volume effects, noise, and other imaging artefacts, such as movement artefacts, since it has a lower ratio of surface voxels to cortex thickness. On the contrary, trabecular parameters, such as Tb.Th, showed weaker agreement with micro-CT, likely since trabeculae are only a few voxels thick in HR-pQCT images. We also observed moderate to good agreement for samples representing diseased, e.g. osteoporotic, bone.

However, no segmentation method performed equally well for all computational applications. While BV/TV was most precisely measured without microstructural segmentation (i.e. grey-scale BV/TV) for 82 μm HR-pQCT, trabecular parameters, such as Tb.N and Tb.Sp, showed higher precision when using a fixed or local adaptive threshold approach. This was especially true for anatomical sites other than the radius and tibia. In contrast, using a threshold which matched morphological parameters resulted in incorrect predictions of mechanical measures such as stress, apparent stiffness, and failure load criteria (Alsayednoor et al., 2018). Despite the evidence for a more careful selection of segmentation method, most studies used the manufacturer's undocumented approaches for segmentation and morphometric parameter computation. The lack of standardization across studies is likely a major factor as to why newly developed tools are rarely adopted by other research groups. We identified four major issues of standardization that affect our ability to validate new and existing methods and limit their use across research groups.

### 6.1 Inconsistent pre-processing

While micro-CT was consistently used as the gold standard, the pre-processing methods of these images were not standardized. This lack of consistency resulted in noticeable systematic deviations in the regression of morphological parameters, making it impossible to conclude whether these parameters are under- or overestimated by HR-pQCT. Hence, future studies should aim to define standards for pre-processing of both micro-CT and HR-pQCT images.

### 6.2 Limited comparisons to similar methods

Studies that proposed new analysis methods did not always directly compare the results of their approach to those from already existing methods. This was particularly noticeable for FEA studies, where every group used different material models, material properties, geometries, and boundary conditions. The use of independent datasets further complicated the differentiation between method- and dataset-based biases in the results.

### 6.3 Limited validation on heterogeneous datasets

Due to the observed dependency of analysis methods on anatomical site and patient cohort, the results from single studies with homogenous datasets cannot directly be compared to one another and the lack of dataset diversity prohibits sufficient assessment of method robustness. Importantly, if these proposed methods are incorporated into proprietary commercial software, opportunities for external validation are then severely limited. Future method development and validation must therefore be based on more diverse datasets to allow for the differentiation between dataset- and pre-processing effects, as this is currently unclear.

### 6.4 Lack of 61 μm HR-pQCT validation

Based on the limited number of 61 μm HR-pQCT validation studies, morphological agreement with micro-CT was improved compared to 82 μm HR-pQCT. While this improved accuracy in morphometric parameters shows promise for mechanical analysis using HR-pQCT FEA, these analyses have yet to be validated against micro-CT FEA. Unfortunately, the validation of morphological parameters is also incomplete, as several morphological parameters have not yet been validated for 61 μm HR-pQCT. As 61 μm HR-pQCT is expected to outperform other clinical imaging modalities, additional validation studies are urgently needed to ensure widespread clinical use of this advanced imaging technology.

### 6.5 Conclusion

HR-pQCT is a promising technology with a variety of potential clinical applications. The improved resolution of 61 μm HR-pQCT provides superior quantification of bone morphometrics when compared to 82 μm HR-pQCT. However, the clear lack of standardization is prohibitive for widespread clinical use. Despite the number of studies using HR-pQCT, there is little standardization or agreement as to how to calculate many morphometric indices or perform FEA. Results of HR-pQCT studies are difficult to compare due to varied pre-processing methods. While many groups use the manufacturer's recommended pre-processing, studies have already shown that there are more accurate methods for some morphometric parameters (e.g. Tb.N). However, due to the lack of a publicly available, heterogeneous, and comprehensive dataset for methods validation, studies proposing new analysis methods often draw an incomplete picture of their methods' capabilities. As such, adoption and cross-validation of new methods by other groups is slow and often non-existent. With the availability of 61 μm HR-pQCT, the community must learn from its mistakes with the introduction of 82 μm HR-pQCT and establish an open access software and data repository, instead of referencing the conclusions of studies using older generation devices with completely different physical properties. Using this data repository, new methods can be benchmarked against the same comprehensive dataset to allow for straightforward comparison. Optimal analysis methods can be selected for each specific application, and updates to methods and their effects on parameters can be immediately accessed through the online repository. With the ability to properly validate new technologies, we can ensure that the clinical use of HR-pQCT can truly provide value in patient diagnosis and care.

## Declaration of competing interest

The authors declare that they have no competing interests.
